# Effects of a narrative template on memory for the time of movie scenes: automatic reshaping is independent of consolidation

**DOI:** 10.1007/s00426-022-01684-w

**Published:** 2022-05-07

**Authors:** Matteo Frisoni, Monica Di Ghionno, Roberto Guidotti, Annalisa Tosoni, Carlo Sestieri

**Affiliations:** Department of Neuroscience, Imaging and Clinical Sciences, ITAB Institute for Advanced Biomedical Technologies, University G. d’Annunzio of Chieti-Pescara, Via dei Vestini 31, 66100 Chieti, Italy

## Abstract

Memory for time is influenced by reconstructive processes, but the underlying mechanisms remain unclear. The present study investigated whether the effect of schematic prior knowledge on temporal memory for movie scenes, produced by the incomplete presentation (cut) of the movie at encoding, is modulated by cut position, retention interval, and task repetition. In a timeline positioning task, participants were asked to indicate when short video clips extracted from a previously encoded movie occurred on a horizontal timeline that represented the video duration. In line with previous findings, removing the final part of the movie resulted in a systematic underestimation of clips' position as a function of their proximity to the missing part. Further experiments demonstrate that the direction of this automatic effect depends on which part of the movie is deleted from the encoding session, consistent with the inferential structure of the schema, and does not depend on consolidation nor reconsolidation processes, at least within the present experimental conditions. We propose that the observed bias depends on the automatic influence of reconstructive processes on judgments about the time of occurrence, based on prior schematic knowledge.

## Introduction

The way humans organize temporal memories of specific events is related to what they already know about the unfolding of similar, frequently experienced activities (Friedman, 1993, 2004). It is long known that episodic memory retrieval is highly influenced by prior semantic knowledge and shaped by reconstructive processes (Bartlett, 1932; Schacter et al., 2011). While such a cognitive mechanism is thought to serve an adaptive function by increasing the precision of temporal judgments, it is also prone to memory distortions in several domains (Schacter, 1999, 2012). For instance, when being asked to recall their past feelings about a specific event, people tend to adapt them to current circumstances, showing a “consistency bias” (Levine, 1997).

A classical line of research has demonstrated that people are equipped with semantic templates acquired via multiple presentations with narrative experiences that tend to share a general time course (Mandler & Johnson, 1977; Thorndyke, 1984). Memory schemas and scripts (Alba & Hasher, 1983; Bartlett, 1932; Ghosh & Gilboa, 2014; Rumelhart, 1980; Schank & Abelson, 1977) convey the structure of stereotypical situations (i.e., elements involved, semantic and temporal relations among those elements) that allow us to understand, predict, and recall events. When activated, narrative schemas are thought to form a set of inferences about the possible occurrence of specific events within the context of the schema (Anderson, 1984; Ghosh & Gilboa, 2014; Rumelhart & Ortony, 1977; Schank, 1983). Such a role is consistent with the broader idea that our brain is evolved to generate predictions of future scenarios using internal models of the world which are continuously compared with incoming stimuli. When inconsistent information is encountered, these models are updated to minimize prediction errors (Barron et al., 2020; van Kesteren & Meeter, 2020).

Memory schemas serve multiple purposes. For example, they provide an automatic retrieval mechanism to judge the temporal position of story events (Mandler & Johnson, 1977; Manlder, 1978; Stein & Glenn, 1979). Again, this process is not necessarily beneficial, as it can also distort the faithful recall of the narrative time. Previous research on “story grammar” has shown that people are prone to schematic intrusions during the recall of narrative material. They are more likely to retrieve an ill-formed/irregular story in a canonical order including constituents of an ideal story schema and to add portions of the narrative to their recall when a story is told with some of its constituents missing (Kintsch & van Dijk, 1978; Mandler & Johnson, 1977; Rumelhart, 1975; Stein & Glenn, 1979; Thorndyke, 1977).

To describe the temporal features of a narrative template, we borrowed some elements from the notion of “script” (Schank & Abelson, 1977): temporal sequence, schematic prior knowledge, common high-level elements extracted by multiple presentation with similar material, and the automatic capacity to add missing parts to the global representation. Thus, by “script-based prior knowledge” we refer to a general kind of knowledge about typical story components and how narrative events are likely to unfold. Similar to the “restaurant script” which can be adopted to recall a dinner at McDonald’s or a Michelin-star restaurant, this narrative template would be used for a very broad range of stories and their common minimal trajectory. Indeed, the typical structure of movies is of the kind “beginning-middle-end” (Cutting, 2016), and there is evidence that different movies share common “large-scale parts” (Bordwell, 2006; Thompson, 1999), independently from the story genre (Cutting & Armstrong, 2019).

In a recent study, we used a timeline positioning task and complex audiovisual stimuli to investigate the influence of prior knowledge on memory for the time of movie scenes (Frisoni et al., 2021). Participants watched a movie and were later asked to indicate when video clips extracted from the movie occurred on a continuous timeline.

We found that overall performance was highly precise, with an average error of about 9 min (full-length film =  ~ 87 min) and no systematic tendency to overestimate or underestimate the clips' time of occurrence as a function of their position in the story plot. However, removing the final part of the movie from the encoding session resulted in a systematic bias in memory for time. Specifically, participants increasingly underestimated the time of occurrence of the video clips as a function of their proximity to the missing part of the movie, regardless of the specific type of audio-visual material. We argued that this last result reflects the automatic activation of the whole movie schema, which conflicts with the representation of the encoded video, and the consequential intrusion of the missing part into the temporal judgments.

### Manipulation of the missing part

However, our previous study left many open questions. A first issue concerns the fact that the manipulation only affected the last part of the movie. Memory schemas can be thought of as predefined structures composed of empty variables (or slots, or terminals) to be filled with incoming stimuli (Gilboa & Marlatte, 2017; Minsky, 1975; Schank & Abelson, 1977). In this regard, the minimal structure of popular movies could be conceived as having a set of four acts: setup, complication, development, and climax (Bordwell, 2006; Cutting, 2016; Thompson, 1999). Specifically, it is unclear to what extent the temporal distortion is modulated by the specific missing constituent and whether it impacts the direction (i.e., underestimation or overestimation) of the reconstructive bias. We believe that the direction and extent of the bias might reveal important information about the internal structure of the memory schema. Indeed, it is known that schematic elements are not encoded equivalently but are woven into a coherent set of interrelations (Anderson, 1984; Rumelhart, 1980). Thus, the manipulation of the position of the removed movie part allows us to investigate not only the presence of a temporal bias per se (i.e., if it generalizes to different movie parts which are removed from the encoding session) but also the degree of interconnection between the removed part and other schematic elements (Ghosh & Gilboa, 2014).

### Retention interval and memory consolidation

Another open question concerns the role of memory consolidation processes on the observed bias. Memory consolidation refers to the gradual stabilization of mnemonic traces over time (Dudai, 2004; Muller & Pilzecker, 1900; Squire et al., 2015) which makes memories increasingly less vulnerable to amnesic agents or retroactive interferences (Hupbach et al., 2015). It has been hypothesized that new memories become transformed and integrated via modifications of a preexisting schema that represents related pieces of information (McClelland et al., 1995; van Kesteren et al., 2010; Winocur et al., 2010). In other words, as memories become remote, schematization increases. Animal studies have shown that rapid schema-based consolidation occurs within a 24-h interval (Bayley et al., 2005; Runyan et al., 2019; Tse et al., 2007). Thus, one possibility is that no, or only a moderate, underestimation bias is manifested immediately, while the bias becomes evident or increases *over time*, as consolidation promotes the incorporation into a pre-existing schema.

However, other lines of evidence suggest that schematic knowledge leads to *immediate* effects on memory, regardless of the passage of time (Alba & Hasher, 1983). In other words, the effect might instead depend on the direct influence of semantic knowledge over episodic memory during judgments about time-of-occurrence. Prior knowledge exerts a profound impact on event memories both at encoding and retrieval. Accordingly, the Event Horizon Model (Radvansky & Zacks, 2017) proposes that event schemas and event models interact both at the level of encoding and retrieval of long-term memory traces (Grafman et al., 1995; Mandler, 1978; Newberry & Bailey, 2019; Rumelhart & Ortony, 1977). According to this view, people would automatically reconstruct the temporal position of movie scenes by relying on an overarching framework of conventional stories.

### Task repetition and reconsolidation

A final question concerns the degree to which the bias remains stable or increases over multiple executions of the retrieval task. On the one hand, the observation that participants’ performance is especially prone to schematic intrusions when an unusual task has to be carried out for the first time (Mandler & Goodman, 1982) might predict a *reduction* of the schematic bias during task repetition. In our case, this could bring to the hypothesis that performing the task a second time could make the judgments less dependent on schematic scaffolding.

On the other hand, task repetition might even *increase* the temporal distortion due to the effect of memory reconsolidation. Reconsolidation refers to the observation that, after standard consolidation, memory retrieval can return memories to a labile state, from which they are then restabilized (Lee et al., 2017; McKenzie & Eichenbaum, 2011; Nader & Einarsson, 2010). The capability to turn old information into malleable traces might serve to adapt them to new circumstances (Dudai, 2004; Lee, 2009). There is evidence that the repeated recall of stories tends to reshape narrative material according to schematic reconstructions (Bartlett, 1932; Bergman & Roediger, 1999; Johnson, 1962). If reconsolidation enhances schema assimilation, which is “the active process of adapting new external information to fit with internal cognitive structures or schemas” (Gilboa & Marlatte, 2017), repeating the task might lead to a further schematization, according to the typical temporal unfolding of popular movies.

### The present study

Using an experimental paradigm similar to that of our previous work, in the present study we aimed at addressing these outstanding issues by manipulating several experimental variables. Experiment 1 was a replication of the cut-before-end procedure, with modified instructions to rule out the possibility that the temporal distortion reflects a conscious adjustment caused by a misinterpretation of the task and a post-experimental interview to test the intentional use of a schematic representation. We expected to replicate our previous findings of an underestimation bias that reflects the automatic intrusion of the missing part into the temporal representation of the movie.

Experiment 2 aimed at testing whether the bias depends on the specific missing part of the movie, by cutting the *middle* third of the movie. If the bias reflects the automatic activation of the full movie script, then subjects should now incorporate the unseen middle part into the temporal representation of the video. In turn, this should now create an *overestimation* bias for the video clips belonging to the final part of the movie. We might expect a symmetric effect on clips from the first part of the movie, although the asymmetry in the presence of causal antecedents between the initial and final part of the movie (Cutting, 2016) might suggest a lower sensibility of the first part to the cutting procedure.

Finally, Experiment 3 tested the effect of memory consolidation and reconsolidation on the underestimation bias observed in Experiment 1. This time participants were tested both immediately after the encoding session (immediate recall condition) and 24 h later (delayed recall condition, as in Experiment 1). We hypothesized that if the bias depends on memory consolidation, it should be absent or decreased in the immediate recall compared to the delayed recall condition. Instead, if the bias reflects the direct influence of semantic memory over judgments about time-of-occurrence, it should already be present in the immediate recall condition and should not differ across experiments. Furthermore, if repeating the retrieval task modulates the schema assimilation via reconsolidation (i.e., enhanced bias) or practice (reduced bias), then a significant difference in the degree of bias should be observed when comparing the delayed recall condition of Exp. 3 with that of Exp. 1.

## Experiment 1

The first experiment was a replication of our previous study (Frisoni et al., 2021) aiming to test the influence of script-based prior knowledge on temporal memory for movie scenes. Twenty-four hours after the encoding of an episode of the BBC’s show “Sherlock”, participants performed judgments about the time of occurrence on a series of 2 s video clips extracted at different points from the same or a different episode.

As in our original study, participants only encoded the first ~ 2/3 of the full movie, under the assumption that viewing a large part of the film activates a pre-existing schema of the prototypical story, leading to the automatic incorporation of the missing part into the temporal representation of the seen video. As a result, we expected increasing anticipation of clips closer to the missing part of the film. To note, in our previous work (Frisoni et al., 2021) no specific bias was observed in any part of the movie when participants encoded the same episode in full and a similar bias was also observed when participants watched the first 2/3 of a very different movie (i.e. “Wallace & Gromit: The Curse of the Were-Rabbit”), suggesting that the eventual effect of the cut is not driven by the movie content. This time, we further emphasized the instruction to treat the timeline as representing the duration of the presented video (vs. the full movie) and we interviewed participants after the experiment to test the intentional use of the schematic representations.

### Methods

#### Participants

Eighteen volunteers (17 females; aged 19–30; mean age: 21.7 years), with no familiarity with the show, participated in the study. The sample size was based on a power analysis conducted using G*power (Faul et al., 2007) on the effect sizes from our previous study (${\eta }_{\mathrm{p}}^{2}$ = 0.21 for relative error). Participants were unaware of the purpose of the experiment, reported normal or corrected-to-normal vision, and provided informed consent before the experiment following guidelines set by the Human Studies Committee of G. d’Annunzio University of Chieti. The study included an encoding session (duration: 60 min) followed by a retrieval session (duration: approx. 30 min). The sessions were separated by a ~ 24 h interval.

#### Stimuli and procedure

The experimental paradigm was composed of a study phase and a test phase, both performed in a darkened testing room. Stimuli were presented on a 17’ LCD computer monitor (1024 × 768 pixels, 60 Hz refresh rate) at a distance of ~ 60 cm. Subjects wore headphones and listened to the audio track dubbed in Italian at a comfortable level (~ 60 dB). In the encoding session, participants were presented with the first 60-min segment of the BBC’s movie “Sherlock” (Season 1, Episode 1, “A study in pink”; duration: 87:30 min) using VLC media player version 2.20. In the retrieval session, a series of video clips (*N* = 228, 216 for the main experiment, 12 for practice and instructions) of 2 s duration were presented using E-Prime 2.0 software (Psychology Software Tools) in random order across subjects.

Each trial started with the presentation of the video clip at the centre of the screen. Participants performed old/new judgments through a mouse button (index/middle finger of the right hand, respectively). The clip was followed by a red fixation cross (size 0.8° visual angle) until a judgment was given. Stimuli [*N* = 114 (108 + 6)] were uniformly sampled every 30 s (measured from the centre of the clip) from the beginning of the movie but few of the clips were not presented as they were used in a different memory test (data not shown). New stimuli [*N* = 114 (108 + 6)] were selected from another episode of the same show (Episode 2, “The Blind Banker”) with the same temporal criteria. Following old responses, participants provided a memory for time judgment on a central horizontal grey line (size: 0.9° × 19.8° visual angle) made of 720 consecutive segments, each corresponding to 5 s of the movie. Participants selected a point on the line to indicate the clip's time of occurrence. A cursor (arrow pointer) was visible and moved in synchronization with the mouse. The timeline remained on the screen for a maximum of 10 s, followed by an intertrial interval (ITI) of 2 s. No feedback was provided.

Importantly, the instructions made clear that the line represented the duration of the video that was shown in the encoding session and not the full length of the movie. At the end of the experiment, participants were interviewed to test whether the underestimation bias was a conscious/intentional distortion and whether they thought that they had been affected by the missing part of the movie.

#### Data analysis

Recognition memory was calculated as *d* prime (according to signal detection theory; Green & Swets, 1966), while the precision of temporal memory for the correctly recognized old clips (hits) was calculated as the temporal distance, quantified in secs, between the line segment selected by the subject and the correct segment for each trial. False alarms, i.e., position judgements provided for clips that were erroneously identified as old, were not included in the analysis as we considered these judgements as not meaningful for the calculation of a memory temporal positioning error, also given their limited number (false alarms rate was 0.04 across subjects).

A continuous timewise analysis investigated the presence of a significant under- or over-estimation effect across subjects (one-sample *t*-test against 0) for each clip, corrected for multiple comparisons using cluster-level based thresholding (Maris & Oostenveld, 2007; Woo et al., 2014). Specifically, after clustering contiguous points showing a significant effect (*p* < 0.05), a permutation test was performed to obtain a null distribution of cluster size in a random dataset with the same numerosity and calculate the probability of having a cluster of a particular size. A thousand permutations with a random shuffling of data in our dataset indicated that clusters of 4 contiguous timepoints survived correction for multiple comparisons (cluster level *p* < 0.05).

To further quantify variations of performance as a function of the clip position, we split the movie into six equal parts of 10 min and used the part as an independent variable in a 1-way repeated-measures analysis of variance (ANOVAs). This arbitrary division was chosen as the best compromise between temporal sampling and the presence of a sufficient number of clips in each part (*N* = 18). Tukey HSD Post Hoc tests were used to test differences between part pairs. We also conducted a series of one-sample *t*-tests (two-tailed, against zero) with Bonferroni correction to determine the presence of a significant amount of relative error in each movie part, indicating a significant bias across participants. To test whether the precision of memory for time varied as a function of the position of the clips, trial-by-trial Spearman rank order correlation tests were conducted between the group-level relative error for each clip and the corresponding clip distance from the beginning of the presented video (clip onset).

### Results

**Fig. 1:**
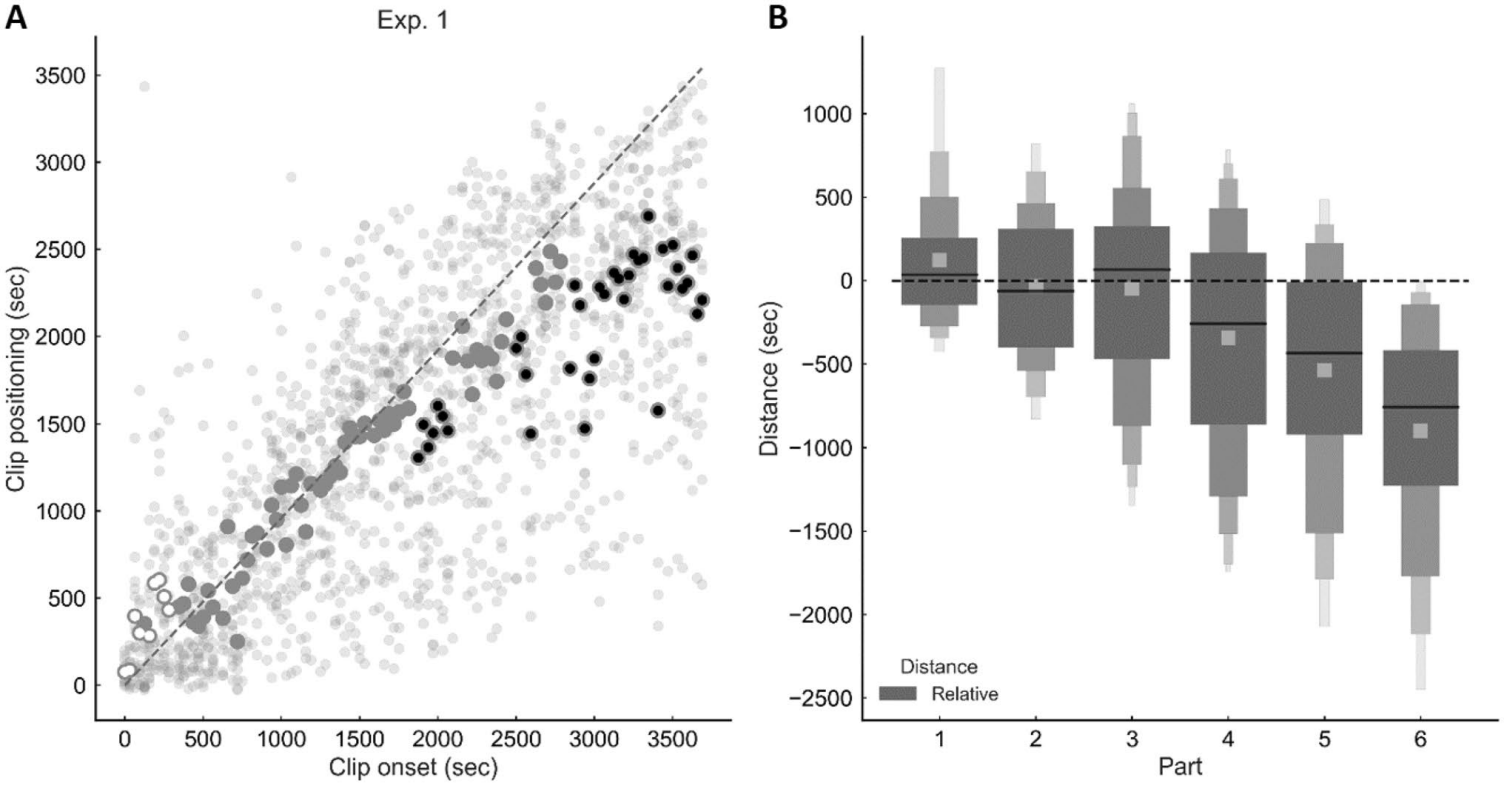
Results of Experiment 1. The figure illustrates the memory for time performance for video clips extracted from the first 60 min of the “Sherlock” episode, which was presented the day before. **A** Scatter plot showing the relationship between actual (*x*-axis) and estimated position (*y*-axis). Circles above or below the diagonal represent an over- and an under-estimation, respectively. Transparent circles indicate single-subject data while opaque circles indicate group data. White and black circles indicate a significant overestimation or underestimation effect, respectively (timewise one-sample *t*-test, corrected for multiple comparisons). **B** Relative error as a function of the video part (part duration: 10 min). The plot shows the group average (lighter square) and the quantiles of the distribution for each part: the bigger box represents the second quartile (Q1–Q3) while smaller boxes represent lower-order quantiles (octiles, hexadeciles, etc.)

Item recognition *d′* was 2.85. The overall level of relative error across trials was − 299 ± 328 s. Figure 1A indicates the presence of a significant overestimation across subjects for some of the clips belonging to the very first section of the presented video and a larger underestimation effect for clips belonging to its final part.

The ANOVA (Fig. 1B) revealed a significant main effect of movie part on relative error [*F* (5, 85) = 27.662, *p* < 0.0001, ${\eta }_{\mathrm{p}}^{2}$ = 0.62]. Post-hoc analyses showed that judgments for clips from Part 1 and Part 2 were significantly more precise compared to those for clips from Part 4 to 6 (all *p* < 0.05). Furthermore, Part 6 was significantly different from Part 1 to 4 (all *p* < 0.001) and Part 5 (*p* < 0.01). Importantly, one-sample *t*-tests revealed that Part 5 and Part 6 (all *p* < 0.0001) were significantly different from zero. Clips that were closer to the last part of the video were strongly associated with an increased level of underestimation (significant negative correlation between relative error and clip onset, *r*_s_ = − 0.89, *p* < 0.0001). An individual-subject analysis demonstrated that the latter Spearman Rank Order correlation was significant (*p* < 0.05) in 16/18 subjects.

Therefore, in line with our original study, the data indicate the presence of an increasing anticipation bias as a function of the clip's distance from the cut. Importantly, none of the subjects reported being aware of the bias or treating the timeline as a representation of the full movie.

### Discussion

Experiment 1 replicated the findings of our original study, confirming that memory for the time of movie scenes is systematically distorted when participants are not allowed to watch the final part of a film with a canonical plot. Importantly, our earlier study ruled out the possibility that the bias was associated with movie content or the manual response. Specifically, when the entire movie was presented at encoding, no bias was observed for any of the movie parts and response distribution was highly homogeneous across the entire timeline (Frisoni et al., 2021). The present results further indicate that the observed systematic bias is likely the effect of the automatic, unintentional activation of a schematic representation rather than of a conscious adjustment of responses and suggest that the reconstructive processes are neither planned nor accessible to awareness (Schacter, 1995). In the following experiment, we tested whether the bias depends on which movie part is removed from the study phase.

## Experiment 2

Experiment 2 investigated the presence of schematic knowledge effects on memory for time by removing a different part of the movie during the encoding phase. Participants were again presented with only 2/3 of the film, but this time the cut involved the middle rather than the final part. In a canonical script, the middle section of the story's macrostructure contains the plot complication and the main actions towards the final resolution/movie ending (Branigan, 1992; Cutting, 2016).

The manipulation might have different effects. One possibility is that the bias is only observed when deleting the last part of the movie and that deleting other parts does not result in a systematic effect. Instead, if the bias reflects the automatic activation of the full movie schema, the internal structure of the schema may be crucial for a specific direction (i.e., underestimation or overestimation) of the bias to occur. This would support the claim that the structure of the movie schema is necessary for reconstructing the clips’ position according to a specific direction. Specifically, removing the middle part of the movie was now expected to produce an *overestimation* effect for clips belonging to the second half of the presented video, since this part should be moved ahead to make room for the unseen middle part. Therefore, the current manipulation should exert the opposite effect on the clips that have to be positioned on the right part of the visual analogue scale compared to those of Experiment 1. In addition, if the elements are equally connected within the schema, removing the middle part would also trigger a symmetrical shift (*underestimation*) on clips taken from the first half of the video.

### Methods

#### Participants

Nineteen new volunteers (18 females; aged 21–26; mean age: 23.2 years) participated in the study, with the same inclusion criteria of Experiment 1.

#### Stimuli and procedure

Experiment 2 was a replication of Experiment 1 with two main differences. First, participants now watched a 60-min version of the first episode of “Sherlock” that combined approximately the first (00:00–29:59 min) and the last (57:31–87:30 min) third of the full-length episode. A 2-s black screen separated the two movie fractions. Therefore, only clips extracted from the first half of the video were the same as those from Part 1–3 in Experiment 1.

The second difference concerns the experimental setup. This time, both sessions were conducted remotely (because of the COVID-19 restrictions), in a web-based version of Experiment 1. The video was sent via the Internet and subjects were asked to watch it only once without pausing. In the retrieval session, the video clips were presented using the online survey platform Qualtrics XM. Participants were instructed to perform both experimental sessions using their laptops in a quiet room and to maintain a distance from the screen of about 60 cm. At retrieval, both the item recognition and the temporal judgments were provided using the touchpad. Although we acknowledge that this procedure gave us less control over the experimental setting, we note that the present findings are not based on measures of reaction times, which are more sensitive to changes in experimental procedures, and that different response devices might potentially result in a general effect over measures of accuracy, rather than in a bias towards a specific direction.

#### Data analysis

Data analysis was similar to Experiment 1. To investigate the effect of the position of the removed movie part on the relative error, we compared memory for time performance in Experiments 1 and 2 using a mixed model ANOVA with the within factor Video Part (1–6) and the between factor Cut Position (Exp1 End, Exp 2 Middle). Moreover, the correlation analysis between relative error and clip onset was performed on the fraction of the presented video that showed a consistent bias.

### Results

**Fig. 2:**
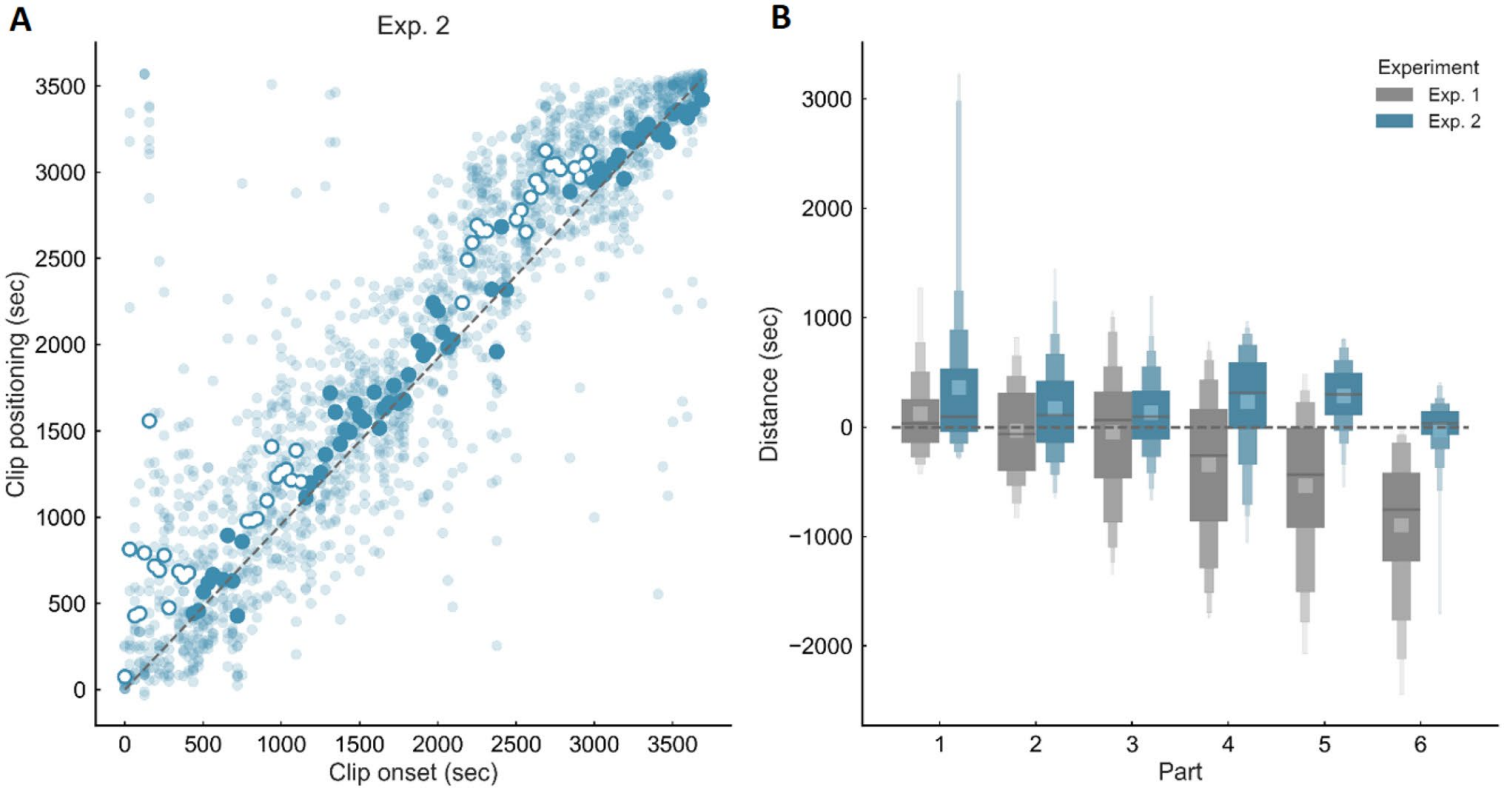
Results of Experiment 2. Memory for time performance for video clips extracted from the “Sherlock” episode, of which only the first and the last thirds were presented at encoding. **A** Scatter plot showing the relationship between actual and estimated position. White circles indicate a significant overestimation effect at the group level. **B** Relative error as a function of the video part in Experiment 1 (grey) and Experiment 2 (blue)

Item recognition *d′* was 3.12. The level of relative error across trials was 187 ± 149 s. In contrast to Experiment 1, Fig. 2A now indicates the presence of a significant overestimation across subjects for clips belonging to both the first and the second half of the presented video.

The ANOVA (Fig. 2B, blue boxes) revealed a significant main effect of movie part on relative error [*F* (5, 90) = 7.99, *p* < 0.0001, ${\eta }_{\mathrm{p}}^{2}$ = 0.31]. Post-hoc analyses showed that judgments for clips from Part 6 were significantly more precise compared to those for clips from Part 1–5 (all *p* < 0.05). Furthermore, judgments for clips from Part 1 were significantly less precise compared to those from Part 3 and Part 6 (all *p* < 0.01). Importantly, one-sample t-tests revealed that Part 1, 4, and 5 were significantly different from zero (all *p* < 0.005) in the direction of an overestimation. A between-subject ANOVA comparing the performance across Exp. 1 (Fig. 2B, grey) and 2 (blue) showed a significant interaction between video part and cut position [*F* (5, 175) = 13.72, *p* < 0.0001, ${\eta }_{\mathrm{p}}^{2}$ = 0.28]. Post-hoc tests indicated the presence of a significant difference across experiments only for clips extracted from Part 4 to 6 (all *p* < 0.0005), which correspond to the middle and the end sections of the whole movie in Experiment 1 and 2, respectively. A Spearman rank order correlation analysis between clip position and degree of overestimation bias further demonstrated that the overestimation bias observed in the second half of the video increased as a function of the proximity to the cut (*r*_s_ = − 0.59, *p* < 0.0001). This correlation was significant (*p* < 0.05) in the majority (14/19) of the subjects.

Taken together, these results indicate the presence of a temporal bias in Experiment 2 that only affected the second half of the presented video with an opposite direction compared to Experiment 1.

### Discussion

The results of Experiment 2 showed that memory for time of movie scenes was influenced also when the middle part was deleted from the movie. Interestingly, we now found an *overestimation* bias for clips taken from the second half of the video, compared to the underestimation bias observed in Experiment 1, consistent with the idea that participants integrate the missing part into the temporal representation of the movie when making judgments about the time of occurrence. These findings suggest that the middle and end parts are tightly interconnected within the narrative script and their omission influences each other. Indeed, the end section of the movie generally contains scenes that are fully comprehensible in the context of the middle section, that is where the bulk of the relevant story predictions are made. Instead, no clear bias was observed for the parts of the video that preceded the cut, a result that might reflect a lower level of inferential processing at the beginning vs. the following sections of the movie. Specifically, we argue that while the beginning section serves the preliminary narrative purpose of setting the stage for the characters and introducing the story (Cutting, 2016), the middle and end parts are reciprocally tied by inferential links which are embedded within the schema and constrain the direction of the bias. A related question raised by the results of Experiment 2 is whether the schematic effect is due to time-dependent processes rather than to immediate reconstructive memory.

## Experiment 3

Using a fixed retention interval of 24 h, our previous experiments left open the possibility that the reshaping of the temporal representation is caused, or exacerbated, by memory consolidation processes. The rationale of Experiment 3 was therefore to test whether the systematic bias produced by the cut-before-end procedure was also observed when the task was performed immediately after the encoding session. If the effect is due to the direct influence of a semantic template on episodic memory—as we expect—it should be already observed in the immediate recall condition. Instead, if the bias depends on consolidation processes that contribute to memory schematization, a reshaping effect should be absent, or reduced, in the immediate recall condition. The experiment further investigated the role of memory reconsolidation on the magnitude of the temporal bias effect under the rationale that if re-accessing memories is subjected to schema assimilation, the underestimation bias should increase as a function of task repetitions. In contrast, if repeating the task makes it more familiar, the opposite effect might be observed.

Experiment 3 used the same cut-before-end procedure of Experiment 1, except that the participants now carried out the task twice using different trials: one immediately after the movie viewing (immediate recall condition) and the other 24 h later (delayed recall condition). The comparison between the immediate recall condition in Experiment 3 and the delayed recall condition of Experiment 1 tested the role of the retention interval, and thus of memory consolidation, while keeping task repetitions constant (non-repeated conditions). The comparison between the delayed recall condition of Experiment 3 and Experiment 1 tested the role of task repetition, and thus the possible role of memory reconsolidation, while keeping the retention interval constant (delayed recall conditions).

### Methods

#### Participants

Eighteen new volunteers (14 females; aged 20–32; mean age: 23.6 years) participated in the study, with the same inclusion criteria as the previous experiments.

#### Stimuli and procedure

Experiment 3 was similar to Experiment 1. The crucial difference was that the recognition and the timeline positioning tasks were performed in two sessions using different trials, one immediately after and one 24 h after the encoding session. Each session contained 228 trials, resulting in a total of 456 trials. While the first stimulus set (*N* = 114) was the same as the one used in Experiment 1, the other stimulus set included 114 clips sampled every 30 s with a delay of 15 s. Stimulus sets were counterbalanced across sessions.

#### Data analysis

Data analysis was the same as in Experiment 1. To isolate the effect of retention interval and task repetition, we implemented a between-subject approach by comparing the results of Experiment 3 with that of Experiment 1. The effect of the retention interval was assessed through a mixed ANOVA with the between-subject factor Interval (Experiment 3: immediate recall, Experiment 1: delayed recall) and the within-subject factor Video Part (Part 1–6). Importantly, task repetition was constant (first) across experiments. The effect of task repetition was assessed through a different mixed ANOVA with the between-subject factor Repetition (Experiment 3: second, Experiment 1: first) and the within-subject factor Video Part (Part 1–6). This time, the retention interval was constant (delayed) across experiments. Finally, we conducted a within-subject test of the combined effect of retention interval and task repetition using a repeated-measures ANOVA with Interval/Repetition and Video Part (Part 1–6) as factors.

### Results

#### Immediate recall condition: effect of retention interval.

**Fig. 3:**
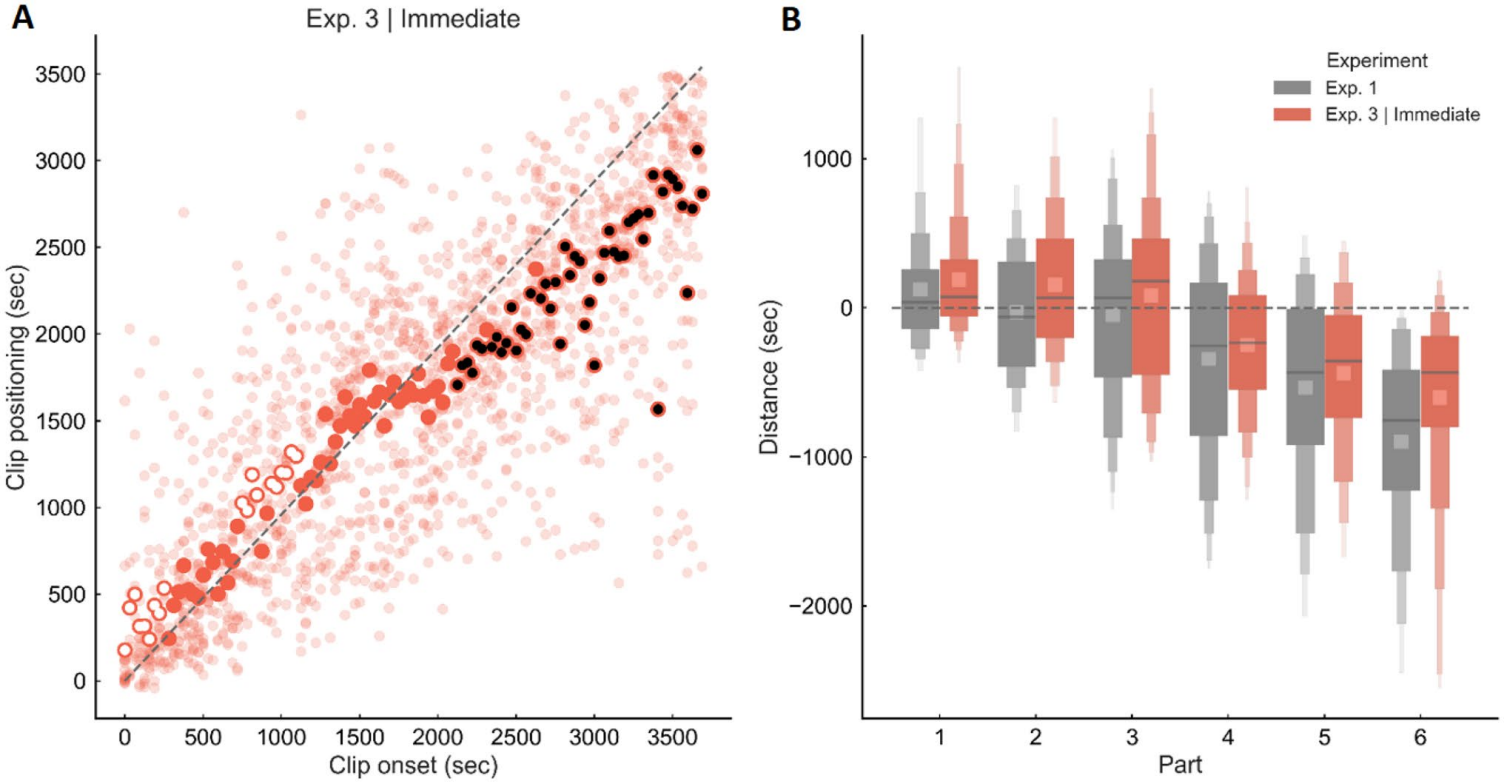
Results of Experiment 3 (immediate recall). Memory for time performance for video clips extracted from the first 60 min of the “Sherlock” episode when the retrieval task was performed immediately after the encoding session. **A** Scatter plot showing the relationship between actual and estimated position. White and black circles indicate a significant overestimation or underestimation effect respectively. **B** Relative error as a function of the video part in Experiment 1 (grey) and Experiment 3, Immediate Recall (red)

Item recognition *d′* was 3.13. The level of relative error across trials was − 153 ± 304 s. Figure 3A shows a similar pattern of results compared to Experiment 1, with a large underestimation effect for clips belonging to the second half of the video. The ANOVA (Fig. 3B, red) revealed a significant main effect of movie part on relative error [*F* (5, 85) = 22.07, *p* < 0.0001, ${\eta }_{\mathrm{p}}^{2}$ = 0.56]. Post-hoc analyses showed that judgments for clips from Part 1 and Part 2 were significantly more precise compared to those for clips from Part 4 to 6 (all *p* < 0.005). Furthermore, Part 6 was significantly different from Part 1 to 3 (all *p* < 0.0005) and Part 4 (*p* < 0.05). Part 5 was significantly different from Part 1 to 3 (all *p* < 0.0005). One-sample *t*-tests revealed that Part 5 and Part 6 (all *p* < 0.0005) were significantly different from zero. Clips that were closer to the last part of the video were associated with an increased level of underestimation (significant negative correlation between relative error and clip onset, *r*_s_ = − 0.85, *p* < 0.0001).

To directly test for a specific effect of retention interval (memory consolidation) we performed a between-experiment comparison (Exp3, immediate recall, vs. Exp1, Fig. 3B; red vs. grey, respectively). The analysis showed no significant main effect of Interval [*F* (1, 34) = 1.98, *p* = 0.18, ${\eta }_{\mathrm{p}}^{2}$ = 0.59] nor a significant interaction between interval and video Part [*F* (5, 170) = 0.89, *p* = 0.49, ${\eta }_{\mathrm{p}}^{2}$ = 0.026].

#### Delayed recall condition: effect of task repetition

**Fig. 4:**
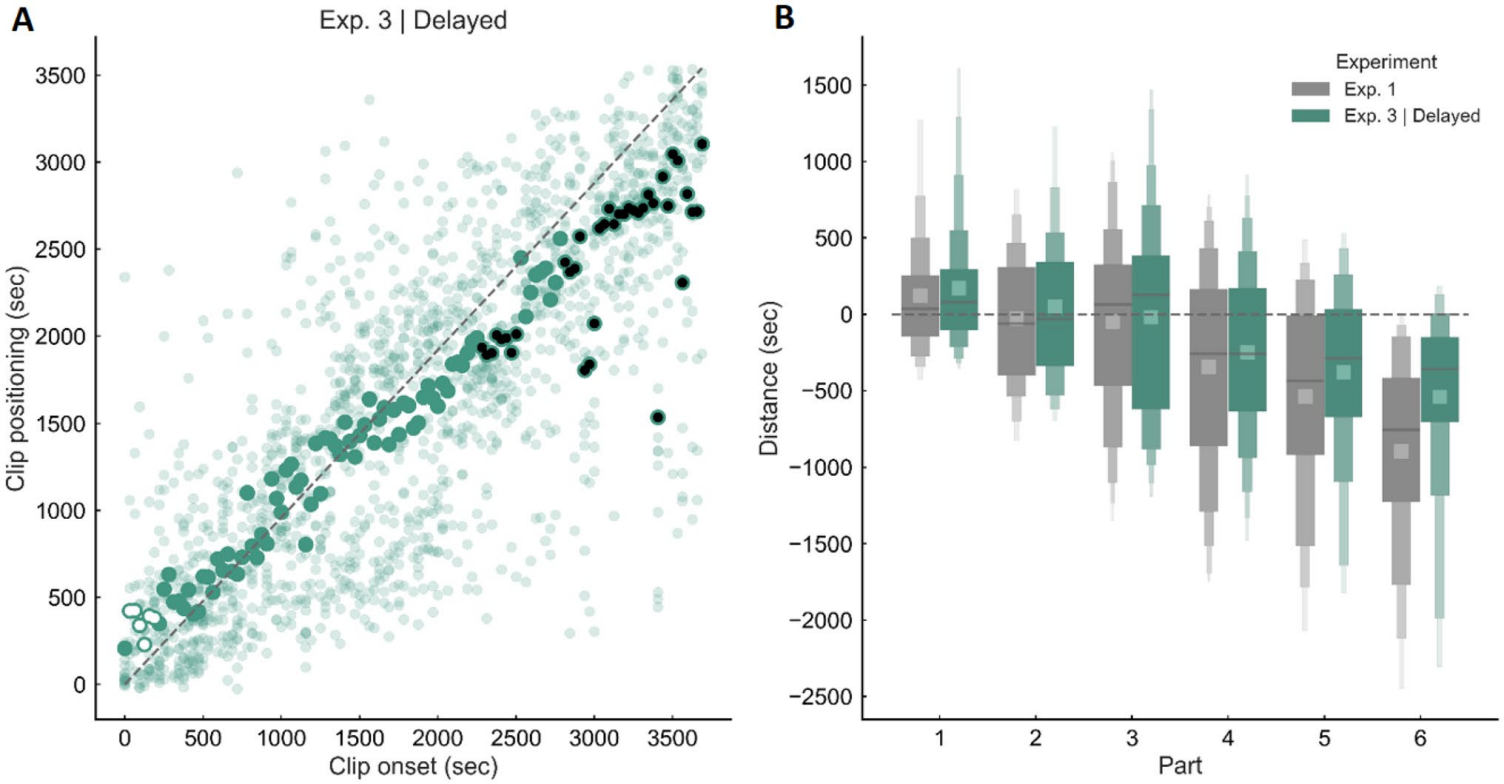
Results of Experiment 3 (delayed recall). Memory for time performance for video clips extracted from the first 60 min of the “Sherlock” episode when the retrieval task was performed a second time the next day. **A** Scatter plot showing the relationship between actual and estimated position. White and black circles indicate a significant overestimation or underestimation effect respectively. **B** Relative error as a function of the video part in Experiment 1 (grey) and Experiment 3, delayed recall (green)

Item recognition *d’* was 3.14. The amount of relative error across trials was − 164 ± 321 s. Figure 4A shows a similar pattern of results compared to Experiments 1 and 3, with a large underestimation effect for clips belonging to the second half of the video. The ANOVA (Fig. 4B, green) revealed a significant main effect of movie part on relative error [*F* (5, 85) = 12.30, *p* < 0.0001, ${\eta }_{\mathrm{p}}^{2}$ = 0.42]. Post-hoc analyses showed that judgments for clips from Part 5 and Part 6 were significantly less precise compared to those for clips from Part 1 to 3 (all *p* < 0.01). Also, judgments for clips from Part 4 were significantly less precise compared to those for clips from Part 1 (*p* < 0.005). One-sample t-tests revealed that Part 5 and Part 6 (all *p* < 0.001) were significantly different from zero. Again, clips that were closer to the last part of the video were associated with an increased level of underestimation (significant negative correlation between relative error and clip onset, *r*_s_ = − 0.79, *p* < 0.0001).

To directly test for a specific effect of task repetition (memory reconsolidation) we performed a between-subject comparison (Exp3 delayed recall vs. Exp1, Fig. 4B, green vs. grey, respectively). The analysis showed no significant main effect of Repetition [*F* (1, 34) = 1.687, *p* = 0.203, ${\eta }_{\mathrm{p}}^{2}$ = 0.047] nor a significant interaction between repetition and video part [*F* (5, 170) = 1.399, *p* = 0.23, ${\eta }_{\mathrm{p}}^{2}$ = 0.040].

#### Within-subject ANOVA—combined effect of retention interval and task repetition.

Finally, we performed a within-experiment comparison to test for a combined effect of retention interval and task repetition using a within-subject design (immediate vs. delayed recall condition). The ANOVA showed no significant main effect of Interval [*F* (1, 17) = 0.08, *p* = 0.78, ${\eta }_{\mathrm{p}}^{2}$ = 0.005], but a significant interaction between interval and video part [*F* (5, 85) = 2.94, *p* = 0.02, ${\eta }_{\mathrm{p}}^{2}$ = 0.148]. However, despite the temporal judgments for each part of the Delayed condition being more accurate (closer to zero) compared to the corresponding part of the immediate condition, the Tukey Post-Hoc Test showed that none of these differences were significant (*p* = n.s.).

### Discussion

The results of Experiment 3 showed that the systematic underestimation bias was also observed when the task was performed immediately after the encoding session, and it did not increase as a function of the length of the retention interval. This finding strongly suggests that participants automatically activate a reference template when they make temporal judgments on cinematic material, even in absence of memory consolidation processes. Consistent with reconstructive theories of memory (e.g., Bartlett, 1932; Conway & Pleydell-Pearce, 2000; Loftus, 1993; Ross, 1989; Schacter, 2012; Spiro, 1980), we suggest that participants automatically reconstructed the position of the events with the aid of prior knowledge about the global schema of conventional stories.

Furthermore, the lack of a significant effect of task repetition indicates that the underestimation bias was also unaffected by reconsolidation processes. While the lack of an effect in the mixed ANOVA might reflect a low sensitivity of the between-subject designs, we note that a similar result was obtained when directly comparing the immediate and the delayed conditions in the same subjects. In fact, a closer look at the within-subject analysis suggests that subjects were even less prone to temporal biases when repeating the task the next day. While this finding is more compatible with a repetition-dependent normalization (i.e., bias reduction) rather than a repetition-dependant schematization (i.e., bias increase) process, we cannot exclude the possibility that reconsolidation occurs only after more than one repetition and/or when the retention interval is longer than that employed in the current study (Bartlett, 1932; Bergman & Roediger, 1999; Johnson, 1962).

## General discussion

Using a procedure similar to our original study on the effect of script-based prior knowledge on memory for time (Frisoni et al., 2021), the present study investigates whether the temporal distortion in memory for the time of movie scenes, caused by the conflict between the representation of what has been seen (the movie fragment) and the more general representation of the movie schema, is automatic (Experiment 1) and whether it varies as a function of the cut position (Experiment 2), retention interval, and task repetition (Experiment 3). First, we replicated the original finding of a systematic underestimation in memory for time when removing the end part of the movie from the encoding session, further demonstrating its automaticity (Experiment 1). For the first time we showed that a memory distortion is also caused by the removal of the middle part of the movie (Experiment 2), but this time participants’ judgments exhibited an *overestimation* bias, which was only observed for clips belonging to the second half of the video. Finally, we provide novel evidence that the bias is also present when the task is performed immediately after the encoding session (Experiment 3) and does not increase as a function of the retention interval, suggesting that consolidation processes are not necessary for its manifestation. The results of the last experiment further indicate that the temporal bias is not affected by task repetitions and therefore appears independent of reconsolidation processes, at least under the present experimental conditions.

### Automatic activation of reconstructive mechanisms in memory for time

The presence of a robust underestimation effect immediately after the encoding phase is consistent with reconstructive models of memory (Bartlett, 1932; Conway & Pleydell-Pearce, 2000; Loftus, 1993; Neisser, 1967; Nelson & Fivush, 2004; Ross, 1989) and with the idea that different aspects of episodic memory are combined with general knowledge of time patterns and conventional locations in time to infer when the event has probably occurred (Friedman, 1993). Specifically, we suggest that participants automatically reconstructed the time of occurrence of the cinematic events drawing upon a global schema of conventional stories.

The independence of the bias from the retention interval further indicates the pervasiveness of the schematic reconstruction in memory for time. This is consistent with a large body of work showing that people have little control over the intrusion of schematic knowledge at retrieval (e.g., addition of missing parts, reordering of events; Kintsch & van Dijk, 1978; Mandler & Johnson, 1977; Rumelhart, 1975; Stein & Glenn, 1979; Thorndyke, 1977). We propose that the present effect might have been emphasized by a sort of “short circuit” caused by the combination of two processes at work. First, participants were asked to position a local event (clip) relative to a global pattern (timeline). This would have probably triggered the most reliable global representation at hand, i.e., the story schema, which is implicitly acquired by extracting statistical regularities from multiple presentations with narrative stories (Gilboa & Marlatte, 2017). In other words, the visual analogue scale might have elicited a global representation of the whole story (that is, a temporal pattern; Friedman, 1993) and strengthened the contribution of reconstructive processes, which taps on knowledge of structured temporal patterns (Friedman, 1993, 2004). Second, removing a meaningful section of the story structure from the movie would elicit a conflict between the partial/incomplete presented video and the activated global schema of the movie. In other words, while the positioning timeline task would trigger the schematic activation, the cutting procedure would lead to the observed schematic effect.

### Temporal bias provides cues about the organization of story schemas

We observed a temporal effect also when removing the middle part from the encoding session. This was expected based on the notion that schemas are structured around different and interconnected units (Alba & Hasher, 1983; Gilboa & Marlatte, 2017; Thorndyke, 1977) allowing one to incorporate missing elements on a large scale through mnemonic reconstruction. While our previous study showed that beginning and end parts constitute crucial temporal landmarks for the schematic reconstruction of narrative time (Frisoni et al., 2021), the results of Experiment 2 may further indicate that temporal memory depends on how elements within the template (in this case middle and end parts) are reciprocally interconnected.

Although cognitive schemas can be conceived of as “filing systems into which specific memories can be put”, they are also actively engaged in the memory process as “integral parts of the memories themselves” (Neisser, 1967). In line with this latter view, a key function of the story schema may be to provide a set of inferences/expectations about what might be coming next and its place in a narrative pattern. The typical expectations would be statistically extracted from multiple presentations with the material (Gilboa & Marlatte, 2017) and embedded within the inferential structure of the schema by forging preferential connections between story elements. The end section of the movie generally contains scenes that are fully comprehensible in the context of the previous section, which is where relevant story predictions are made (Cutting, 2016). Thus, a higher-level interdependency between these two parts might be necessary to better understand the story.

Taken together, these results might indicate that the strong bias in the proximity of the missing end part of the movie is due to long-term inferences that spread beyond the current (and the next) event model. This view would be also consistent with the idea that participants not only recall the clip, but also the associated inferences they have made during encoding (Alba & Hasher, 1983). This might explain why the underestimation effect is stronger for clips that are closer to the missing part, to make room for a larger/growing set of expectations.

### Narrative organization and story schemas

A large body of work from the social sciences has focused on the narrative organization and its effect on the recall of stories (Bruner, 1987, 1991; László, 2014). From a cultural perspective (László, 2008; Pléh, 2018), “narrative thought” (Bruner, 1987) serves the construction of a coherent representation of the world, which can be socially transmissible (Bruner, 1991, 1997; Colby, 1975; Schank & Abelson, 1977). Analogously, the “lived time” (Bruner, 1987) and the “self” (Dennett, 1990, 1992) would be constructed through life stories (Pléh, 2003). In this view, narrative comprehension would be based on social knowledge about the usual motives of human actions. Thus, the interpretation of characters’ intentions would allow the construction of a coherent causal chain, and thus the representation of a specific story structure (Pléh, 2018; Graesser, 1992; Schank, 1975; Schank & Abelson, 1977). Direct measures of online construction (Graesser, 1992, 1996; Graesser & Clark, 1986) can better explain memory for stories than “formal/schematic models” (Black & Bower, 1980; Pléh, 1987).

In our view, however, some major distinctions might be made between the traditional social science literature that directly relates the organization of narrative to the interpretation of intentional actions in narratives and causal relations between events (Radvansky & Zacks, 2017) and our approach. First, we were primarily interested in the fine-grained precision of memory for time, and on how this can be implicitly affected by reconstructive processes, rather than directly studying story construction with an explicit measure. Secondl we believe that the causal chain is highly story-specific, whereas we focused on a more general/pre-existing template that works across different stories following a conventional plot. For example, in our previous study (Frisoni et al., 2021) we showed that the effect of the cut-before-end procedure generalizes for a completely different movie (Wallace & Gromit, in which characters, objects, and background were made of plasticine clay). We further speculate that the causal chain might be especially important when assessing memory for temporal order, i.e., the relations between events (Friedman, 1993, 2004), which is not necessarily associated with precision of memory for the time, as we observed in our previous study (Frisoni et al., 2021).

### Temporal bias is held constant across task repetitions

The analysis of the Delayed condition of Experiment 3 suggests that the underestimation bias is not affected by reconsolidation processes. On the one hand, the null result might reflect the specific influence of the immediate task on subsequent task repetitions. In particular, the immediate recall test might help consolidate a schematically biased material, thereby reducing the possibility of finding further distortion on the successive test. Indeed, there is evidence that distortions or schema-driven intrusions occurring during immediate recall are more likely to be retained on later tests (McDermott, 1996; Roediger et al., 1996; Schooler et al., 1988). On the other hand, it is also possible that more repetitions and/or a larger interval between successive tests are needed to obtain substantial changes in the schematic representation of the same material (Bartlett, 1932; Bergman & Roediger, 1999; Johnson, 1962). For example, previous studies indicate the presence of schematization effects over periods of days (Bergman & Roediger, 1999) and have further shown that schematic errors are greater at longer vs. shorter retention intervals (Sulin & Dooling, 1974).

However, some findings from the present study point toward the presence of subtle differences in the delayed condition that appear to go in the opposite direction (bias reduction). These results might reflect a general weakening of the schematic influence on the retrieved material, which then becomes less prone to a systematic distortion. In this view, the strong reliance on a schematic template when the retrieval task immediately follows the encoding of the movie would be caused by the fact that participants are asked to perform an unusual task for the first time and they need a sort of scaffolding to maximize their performance. Some evidence for this account is represented by the observation that memory distortions are more likely when participants are encouraged to go beyond the limits of what they can effectively recall (Baddeley et al., 2015; Neisser, 1988).

Another tempting possibility is that the decrease of temporal distortion may be due to schema accommodation, that is the adaptation of existing schemas to take new information into account (Gilboa & Marlatte, 2017; Piaget, 1952). This lies at the heart of the idea that schemas are active templates that can change to cope with new situations and progressively form an updated set of inferences. Future experiments will have to determine how and whether memory for time is transformed both by the passage of time and the repeated retrieval of the filmed narratives.

### Limitations and open questions

One could argue that the observed bias is not necessarily caused by what we called script-based prior knowledge. An alternative explanation, in terms of a general violation of expectancy, is that subjects automatically perceive that “something is missing” whenever the video is interrupted. However, an explanation of the bias in terms of an automatic reaction to the interruption of the video would predict a similar effect regardless of the presented material. Contrary to this prediction, our previous study showed that the “cut-before-end procedure” resulted in a different amount of bias depending on the specific movie plot, suggesting that the bias reflects the presence of a movie representation (Frisoni et al., 2021). In theory, also the interruption of a 90 m video showing a man running on the treadmill would still constitute a perceptual violation. However, we believe that in this case it is unlikely that people will add a missing part to their memories, as they would not be able to create a clear trajectory or “parabolic path” (e.g., beginning, middle and end points). Unfortunately, testing temporal precision on *non-narrative material* is not straightforward, as temporal memory performance is supposed to inevitably decay in absence of a meaningful pattern.

A further issue concerns the degree to which the specific movie content affected the observed temporal distortions. Indeed, there is evidence that judgments about event duration are influenced by the number and the degree of similarity between sub-events (Faber & Gennari, 2015, 2017), and that familiar events also have associated typical durations in semantic memory (Coll-Florit & Gennari, 2011). These fine-grained factors might have also contributed to the observed bias. However, more research is needed to establish the specific contribution of such semantic components in modulating the temporal distortion.

It is also possible to hypothesize that the observed bias might have arisen from the imagination of what might have happened in the part of the movie that was not shown (i.e., inferential processes which are associated with the construction of the specific storyline or a sketch of the plot), and that these imagined events influence the estimated time of the events they were actually shown. This interpretation is not far from to the notion of a general narrative template but focus more specifically on the idea that viewers' time estimates are influenced by time in the story world. However, it is difficult to distinguish between the two hypotheses since they make the same predictions in the present context. One way to test for the effect of imagined events would be to investigate temporal judgments in stories containing large or small temporal jumps (see Zwaan, 1996). In this case, the effect of a middle cut should be dependent on the (imagined) story time elapsed in the cut.

## Data statement

Data are available at the following link: Reserved https://doi.org/10.17632/x22vmcnp6c.1.
